# Pixel-by-pixel autofluorescence corrected FRET in fluorescence microscopy improves accuracy for samples with spatially varied autofluorescence to signal ratio

**DOI:** 10.1038/s41598-023-30098-w

**Published:** 2023-02-20

**Authors:** István Rebenku, Cameron B. Lloyd, János Szöllősi, György Vereb

**Affiliations:** 1 Department of Biophysics and Cell Biology, Faculty of Medicine, University of Debrecen, Egyetem tér 1, Debrecen, 4032 Hungary; 2 ELKH-DE Cell Biology and Signaling Research Group, Faculty of Medicine, University of Debrecen, Egyetem tér 1, Debrecen, 4032 Hungary; 3 Faculty of Pharmacy, University of Debrecen, Egyetem tér 1, Debrecen, 4032 Hungary

**Keywords:** Fluorescence imaging, Software, Confocal microscopy, Supramolecular assembly

## Abstract

The actual interaction between signaling species in cellular processes is often more important than their expression levels. Förster resonance energy transfer (FRET) is a popular tool for studying molecular interactions, since it is highly sensitive to proximity in the range of 2–10 nm. Spectral spillover-corrected quantitative (3-cube) FRET is a cost effective and versatile approach, which can be applied in flow cytometry and various modalities of fluorescence microscopy, but may be hampered by varying levels of autofluorescence. Here, we have implemented pixel-by-pixel autofluorescence correction in microscopy FRET measurements, exploiting cell-free calibration standards void of autofluorescence that allow the correct determination of all spectral spillover factors. We also present an ImageJ/Fiji plugin for interactive analysis of single images as well as automatic creation of quantitative FRET efficiency maps from large image sets. For validation, we used bead and cell based FRET models covering a range of signal to autofluorescence ratios and FRET efficiencies and compared the approach with conventional average autofluorescence/background correction. Pixel-by-pixel autofluorescence correction proved to be superior in the accuracy of results, particularly for samples with spatially varying autofluorescence and low fluorescence to autofluorescence ratios, the latter often being the case for physiological expression levels.

## Introduction

Förster resonance energy transfer (FRET) is a non-collisional non-radiative energy transfer between a fluorescent donor dye and a spectrally adequate acceptor dye, which, for practical purposes, is often chosen to be fluorescent^1^. Starting from the late 1960s, FRET has gradually become a popular tool for determining proximity between macromolecules. The efficiency of energy transfer (E) is a function of the inverse sixth power of the distance between the donor and acceptor dyes, which changes rapidly in the range of 2–10 nm. This range of sensitivity serves as the basis for establishing and comparing molecular level interactions even in optical instruments that are otherwise limited to lesser resolving power owed to diffraction^2^. Even with the rapidly improving superresolution techniques pushing the resolution limit lower and lower, plenty of space has remained for FRET techniques^3^. There are a wide variety of approaches to measure FRET in microscopy^4^, from single molecule measurements^5,6^ to ensemble approaches, many of which do not require expensive instrumentation, and/or are non-destructive to the sample^7–9^. These encompass ratiometric methods that are best used in the ever expanding field of fluorescent protein-based biosensors with known donor/acceptor stoichiometry^10^ as well as quantitative measurement modalities that yield calibrated FRET efficiency independent of donor/acceptor stoichiometry^11^. Of these, fluorescence lifetime imaging (FLIM) represents an intrinsically quantitative method but requires advanced instrumentation^12,13^, while spectral spillover-corrected (or three-cube) FRET is a cost effective and versatile approach, which can be applied in flow cytometry and conventional fluorescence microscopy. As opposed to the also popular acceptor photobleaching technique^14,15^, it can also be used in conjunction with time-lapse and 3D image analysis^11,16^.

The main limitation in every method for FRET measurements is the signal to noise ratio (SNR). SNR is the expected value of the signal divided by its SD. Here, the noise of all measured fluorescence intensities used in further calculations propagates into the FRET efficiency. Assuming a Poissonian distribution of detected photons per pixel with expected value λ, if systematic noise is negligible SNR is √λ, so lower intensities are expected to increase the uncertainty of the determined FRET E to a greater extent. Samples with low SNR can be made more amenable to FRET analysis by improving the quantum efficiency and photostability of the fluorescent dyes, by using more efficient detection^17^, optimizing the FRET dye pairs^18,19^, and by improved mathematical and statistical approaches^20–23^. Using the spectral spillover-corrected method generally allows for concluding about existing molecular interactions when a FRET efficiency of ~ 5% or above is seen.

The achieved SNR is also limited by cellular autofluorescence. Since autofluorescence is less pronounced towards the red spectral region, red-shifted donor acceptor dye pairs should preferably be used, such as Alexa Fluor 546–Alexa Fluor 647^24^. Several other methods are also available to decrease the distorting effect of autofluorescence. Spillover of autofluorescence into label fluorescence was corrected for both in microscopy^25^ and in flow cytometry, which greatly improved the accuracy of FRET E determination^26^. Silas Leavesley’s lab has further improved the approach by introducing spectral unmixing^27^ and demonstrated that the method is applicable to assess compartmentalized signaling mechanisms using 3D spectral imaging^28^. In a highly innovative approach, Cardoso Dos Santos et al. have applied time-gating to fluorescence lifetime imaging microscopy and combined quantum dots with fluorescent proteins to assess FRET while excluding native fluorescence^29^. While spectral and lifetime imaging are gaining space, it is the conventional confocal microscope that almost all biologists have access to nowadays. With this in mind, we have set out to improve upon our previous ImageJ/Fiji plugin (RiFRET^16^) for evaluating spectral spillover-corrected FRET measurements by introducing the option of pixel-by-pixel autofluorescence correction. Since the measurement of the instrument calibration factor α is hindered by various uncontrollable circumstances in cellular systems and has only been achieved reliably for fluorescent protein constructs^30,31^, first we have created and validated cell-free standard slides for its accurate determination. These standards also allowed the accurate determination of spectral spillover factors by obviating the confounding effect of cellular autofluorescence. Finally, using a set of models with varying levels of fluorescent signals and autofluorescence, we have demonstrated that investigation of FRET on cells with normal expression level of the proteins of interest and with spatially varying levels of autofluorescence benefits most from pixel-by-pixel autofluorescence correction. The RiFRET v2 ImageJ plugin created for a user-friendly analysis of these measurements is available to the research community via GitHub as well as through the Fiji updater and also allows for the fully automatic analysis of large datasets.

## Results and discussion

### Algorithm for pixel-by-pixel autofluorescence correction

For a pixel-by-pixel corrected FRET measurement four fluorescent channels should be measured: *I*_0_ is the autofluorescence measured in an auxiliary channel which in our example is blue-shifted compared to the donor channel. It should also be possible to use a far red channel for this purpose if the donor and acceptor were more blue-shifted should it not be practical to use near-UV excitation and blue emission (e.g. in the case of using a nuclear stain in this spectral domain). *I*_1_ is the donor channel, which should be spectrally appropriate for the donor dye. *I*_2_ is the transfer channel with donor excitation and acceptor detection range. *I*_3_ is the acceptor channel with excitation and detection suitable for the acceptor dye. This spectral setup makes the method suitable for Alexa Fluor 488/546 and Alexa Fluor 546/647 FRET pairs or other dye pairs with similar spectral properties. The first step of the evaluation is always the subtraction of instrument background for each channel. This needs to be measured on a non-fluorescent slide.

Equations (1–4) below describe the contribution of various components to each fluorescence channel. *AF* is the autofluorescence in channel 0. *I*_*D*_ and *I*_*A*_ are the fluorescence intensity of the unquenched donor and of the acceptor in their own detection channels, respectively. The other components are calibration factors described below.

1 $${I}_{0}=AF+{I}_{D}\left(1-E\right)\cdot {S}_{5}+{I}_{A}\cdot {S}_{6}+{I}_{D}\cdot E\cdot \alpha \cdot \frac{{S}_{6}}{{S}_{2}}$$

2 $${I}_{1}=AF\cdot {B}_{1}+{I}_{D}\left(1-E\right)+{I}_{A}\cdot {S}_{4}+{I}_{D}\cdot E\cdot \alpha \cdot \frac{{S}_{4}}{{S}_{2}}$$

3 $${I}_{2}=AF\cdot {B}_{2}+{I}_{D}\left(1-E\right)\cdot {S}_{1}+{I}_{A}\cdot {S}_{2}+{I}_{D}\cdot E\cdot \alpha$$

4 $${I}_{3}=AF\cdot {B}_{3}+{I}_{D}\left(1-E\right)\cdot {S}_{3}+{I}_{A}+{I}_{D}\cdot E\cdot \alpha \cdot \frac{1}{{S}_{2}}. epsR$$

5 $$epsR=\frac{{\varepsilon }_{A\cdot exc}^{D}\cdot {\varepsilon }_{D.exc}^{A}}{{\varepsilon }_{D\cdot exc}^{D}\cdot {\varepsilon }_{A\cdot exc}^{A}}$$

The *eps*R (Eq. 5) is the product of the ratios of the donor and acceptor dye’s molar absorption coefficients at each other’s excitation wavelengths to their absorption coefficients at their own excitation wavelengths. For most dye pairs this numeric is negligibly small.

*S*_1_, *S*_3_ and *S*_5_ are determined using a sample containing only the donor dye as described in Eqs. (6–8).

6 $${S}_{1}=\frac{{I}_{2}^{D}}{{I}_{1}^{D}}$$

7 $${S}_{3}=\frac{{I}_{3}^{D}}{{I}_{1}^{D}}$$

8 $${S}_{5}=\frac{{I}_{0}^{D}}{{I}_{1}^{D}}$$

*S*_2_, *S*_4_ and *S*_6_ are determined on an acceptor only sample (Eqs. 9–11).

9 $${S}_{2}=\frac{{I}_{2}^{A}}{{I}_{3}^{A}}$$

10 $${S}_{4}=\frac{{I}_{1}^{D}}{{I}_{3}^{A}}$$

11 $${S}_{6}=\frac{{I}_{0}^{A}}{{I}_{3}^{A}}$$

*B*_1_, *B*_2_ and *B*_3_ are determined on unlabeled cellular samples (*Nl*) (Eqs. 12–14).

12 $${B}_{1}=\frac{{I}_{1}^{Nl}}{{I}_{0}^{Nl}}$$

13 $${B}_{2}=\frac{{I}_{2}^{Nl}}{{I}_{0}^{Nl}}$$

14 $${B}_{3}=\frac{{I}_{3}^{Nl}}{{I}_{0}^{Nl}}$$

The alpha parameter corrects for the difference in the quantum efficiency *Q*_*A*_ and *Q*_*D*_ and the detection efficiency *η*_*A*_ and *η*_*D*_ of the donor and the acceptor, respectively, and can be determined on donor and acceptor labeled samples (Eq. 15). When using labeled cells for this purpose, the ratio of expression levels of donor and acceptor molecules should be constant across the measured cells. Ideally, the same epitope is used, targeted once with donor and once with acceptor labeled antibodies. ${I}_{2}^{D}$ and ${I}_{3}^{A}$ are to be measured on these samples and averaged for at least 5–10 images. *L*_*D*_ and *L*_*A*_, are the mean number of dye molecules attached to the donor and the acceptor conjugated antibody, *B*_*D*_ and *B*_*A*_ are the mean number of receptors per cell labeled by the donor and the acceptor conjugated antibody, and ${\varepsilon }_{D}$ and ${\varepsilon }_{A}$ are the molar absorption coefficients of the donor and acceptor dyes at the wavelength of donor excitation.

Alpha can also be determined using standard slides made with the labeled antibodies, in which case *B*_*D*_ and *B*_*A*_ are the molar concentration of the donor and acceptor conjugated antibodies.

15 $$\alpha =\frac{{Q}_{A}}{{Q}_{D}}\cdot \frac{{\eta }_{A}}{{\eta }_{D}}=\frac{{I}_{3}^{A}}{{I}_{2}^{D}}\cdot \frac{{B}_{D}}{{B}_{A}}\cdot \frac{{L}_{D}}{{L}_{A}}\cdot \frac{{\varepsilon }_{D}}{{\varepsilon }_{A}}$$

FRET efficiency is calculated as

16 $$E=\frac{A}{\alpha \cdot \left(epsR-1\right)\cdot \left[{B}_{2}{I}_{0}{S}_{4}+{B}_{1}{I}_{2}{S}_{6}+{I}_{1}\left({S}_{2}-{B}_{2}{S}_{6}\right){-B}_{1}{I}_{0}{S}_{2}-{I}_{2}{S}_{4}\right]+A}$$

where

17 $$\begin{aligned}A&={S}_{2}\left\{{B}_{2}{I}_{0}+{I}_{1}{S}_{1}+{I}_{3}{S}_{2}-{S}_{2}\left({B}_{3}{I}_{0}+{I}_{1}{S}_{3}\right)+{B}_{1}{I}_{0}\left({S}_{2}{S}_{3}-{S}_{1}\right)+\left({B}_{3}{I}_{0}-{I}_{3}\right){S}_{1}{S}_{4}\right. \\&\quad +\left({B}_{3}{I}_{1}-{B}_{1}{I}_{3}\right)\left({S}_{2}{S}_{5}-{S}_{1}{S}_{6}\right)+{I}_{2}\left[{B}_{1}{S}_{5}-{{B}_{3}S}_{4}{S}_{5}+{B}_{3}{S}_{6}+{S}_{3}\left({S}_{4}-{B}_{1}{S}_{6}\right)-1\right]\\ & \quad \left. -{B}_{2}\left[{I}_{0}{S}_{3}{S}_{4}+{I}_{3}\left({S}_{6}-{S}_{4}{S}_{5}\right)+{I}_{1}\left({S}_{5}-{{S}_{3}S}_{6}\right)\right]\right\}\end{aligned}$$

Using this FRET efficiency value, the actual donor, acceptor and autofluorescence values can also be calculated:

18 $${I}_{D}=\frac{1}{1-E}\cdot \frac{{I}_{0}\cdot {B}_{1}-{I}_{1}}{{B}_{1}\cdot {S}_{5}-1}$$

19 $${I}_{A}=\frac{{I}_{0}\cdot {B}_{3}-{I}_{1}\cdot {B}_{3}\cdot {S}_{5}+{I}_{3}\cdot ({B}_{1}\cdot {S}_{5}-1)}{{B}_{1}\cdot {S}_{5}-1}$$

20 $$AF=\frac{{I}_{1}\cdot {S}_{5}-{I}_{0}}{{B}_{1}\cdot {S}_{5}-1}$$

For calculating FRET without pixel-by-pixel autofluorescence correction, the same equations can be used but the *S*_5_, *S*_6_ and *B* factors assume a value of 0. If no autofluorescence correction is done, only the instrument background is subtracted in each image and channel (IBG corrected evaluation). The classical option, however, is correcting with average autofluorescence (AVG corrected evaluation). For this approach, the average autofluorescence for channels *I*_1_, *I*_2_ and *I*_3_ needs to be measured on unlabeled samples using the same instrument settings. These values (after correcting for instrument background) are subtracted as constants from the respective I values instead of using $AF\cdot {B}_{1}$, $AF\cdot {B}_{2}$ and $AF\cdot {B}_{3}$ in Eqs. (2–4). Equation (1) is not used, and calculation of FRET efficiency is simplified to

21 $$E=\frac{{S}_{2}\left({I}_{1}{S}_{1}+{I}_{3}{S}_{2}-{S}_{2}{I}_{1}{S}_{3}-{I}_{3}{S}_{1}{S}_{4}+{I}_{2}{S}_{3}{S}_{4}-{I}_{2}\right)}{\alpha \cdot \left(epsR-1\right)\cdot {(I}_{1}{S}_{2}-{I}_{2}{S}_{4})+{S}_{2}\left({I}_{1}{S}_{1}+{I}_{3}{S}_{2}-{S}_{2}{I}_{1}{S}_{3}-{I}_{3}{S}_{1}{S}_{4}+{I}_{2}{S}_{3}{S}_{4}-{I}_{2}\right)}$$

### Characteristics of cellular autofluorescence

**Figure 1:**
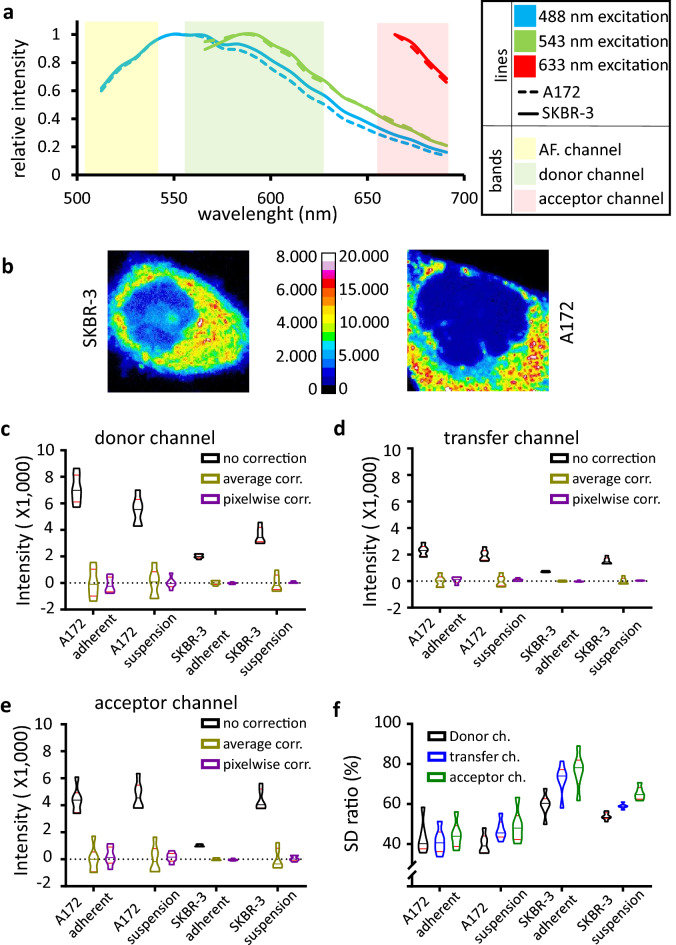
Characterization of cellular autofluorescence and different autofluorescence correction methods. (**a**) Autofluorescence emission spectra of A172 and SKBR-3 cells measured with a spectral detector upon 488; 543 and 633 nm excitation. The typical emission channels of a FRET measurement are represented as colored bands. (**b**) Autofluorescence intensity distribution of an SKBR-3 and an A172 cell. (**c**–**e**) Autofluorescence contribution to the donor, transfer and acceptor channels of a typical FRET measurement before correction, after correction with average autofluorescence and after pixel-by-pixel correction, demonstrated using non-labeled cells grown on a coverslip, as well as cells mounted in suspension after trypsinization. Average emission intensities of 6 independent samples in arbitary units as violin plots (**c** donor channel, **d** transfer channel, **e** acceptor channel). (**f**) Ratio of the standard deviations of pixel-by-pixel corrected to average corrected autofluorescence distributions from data in panels (**c**–**e**).

For testing the autofluorescence correction algorithm, first we have determined the autofluorescence spectra of A172 and SKBR3 cells both when attached to glass surface and in suspension. The spectra of the cell lines show only slight differences through most of the visible spectra (Fig. 1a). Trypsin treatment did not significantly change the autofluorescence properties (Supplementary Fig. 1a). The spectral homogeneity of autofluorescence in the cell lines suggests that it is possible to apply an auxiliary autofluorescence channel for the pixel-by-pixel autofluorescence (PBP AF) correction of the signal in other fluorescence channels, and this channel can use excitation and emission wavelengths/wavelength ranges that are distinctly different from those used for FRET E calculation. This would represent an improvement upon the earlier flow cytometry approach^26^, where excitation of the autofluorescence, donor and transfer channels were at the same wavelength. We have also studied the subcellular distribution of autofluorescence (Fig. 1b), which showed high spatial inhomogeneity of emission intensity. As opposed to the high coefficient of variation and bimodal intensity distribution in every fluorescence channel, the pixelwise distribution of autofluorescence correction factors, generated by dividing the donor, acceptor or transfer images by the autofluorescence reference image, was monomodal, nearly Gaussian, and exhibited a coefficient of variation less than half of the original images (Supplementary Fig. 1b).

### Autofluorescence correction on non-labeled samples

To demonstrate how effective these AF correction factors are in PBP AF correction and what advantages their use may carry over average autofluorescence subtraction, we first tested and compared non-labeled samples in the fluorescence channels typically used in 3-cube FRET measurements (Fig. 1c–f). The perfect autofluorescence correction on a non-labeled sample would result in zero mean intensity with a small standard deviation in every channel. As expected, the average of integrated cellular intensities was close to 0 in the case of both corrections in all three FRET channels (donor, transfer and acceptor, Fig. 1c–e, respectively) indicating that both methods, on average, remove cellular background fluorescence well. However, the plots also reveal that the average correction approach yields a larger spread of corrected cell-by-cell fluorescence values, so while on average this correction works well, many individual cells are not properly corrected. This is supported by calculating the ratio of SDs for PBP AF to average correction (Fig. 1f) which reveals an improvement by 50–60% in the case of A172 cells and by 20–40% in the case of SKBR3 cells, the difference being attributable to the lower autofluorescence values (and their lower absolute divergence) in the latter. In conclusion, it can be said the PBP AF correction provides the expected, correct average values while greatly improving the precision of determination for each individual cell thereby decreasing sampling variance. This in turn is expected to decrease the variance of derived parameters, such as FRET E, by reducing the error propagated into them from multiple images. It is expected that at the subcellular level similar improvements can be achieved, as will be demonstrated further on using biologically relevant FRET samples.

### Correction factors on antibody standard slides

**Figure 2:**
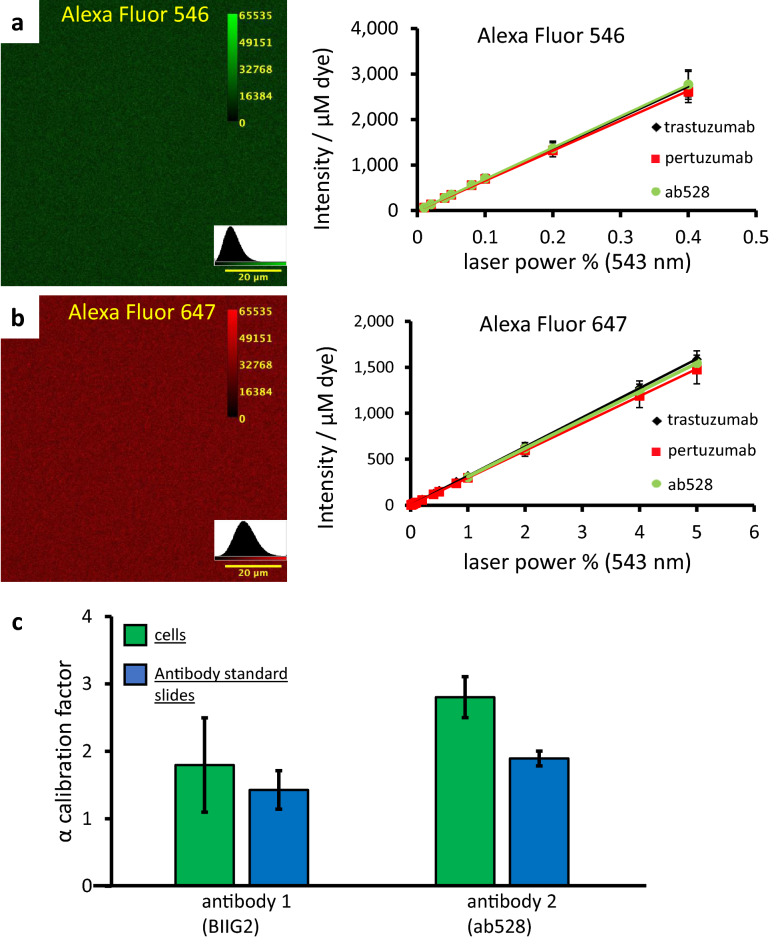
Standard slides for determining the instrument calibration (α) and spectral spillover (S) factors. (**a**,**c**) Images of Alexa Fluor 546 and 647 conjugated trastuzumab film with pixelwise intensity distributions as insets. (**b**,**d**) Average intensities of 3 different antibodies conjugated to Alexa Fluor 546 or Alexa Fluor 647, plotted against 543 nm excitation laser powers, normalized to dye concentration. Error bars represent ± SD of n = 5 images (**c**) α calibration factor determined on A172 cells and on standard slides using two different antibodies. Error bars are ± SD from n = 6 images.

Before proceeding to testing labeled samples, however, a long standing conundrum had to be resolved. In an intensity-based FRET experiment, multiple correction factors have to be determined: spectral spillover factors (S factors, Eqs. 6–11) and the alpha calibration factor (Eq. 15). Every correction factor should be determined on single labeled samples. Because single labeled cellular samples also have their own autofluorescence, which varies on a cell-by-cell and pixel-by-pixel basis, the relative contribution of spectral spillover and autofluorescence to any pixel is unknown, precluding the correct determination of the fluorescence intensities and therefore, of S factors. Thus, we have worked out a recipe to make suitable cell-free calibration slides (see “Materials and methods” section). These slides can be stored for at least up to 6 months at 4 °C without any sign of deterioration. On Fig. 2a,b left panel, we present example images of those samples. We have tested the slides’ performance for S and alpha factor determination. The measured average intensities were normalized to the photometrically determined concentration of the fluorescent dye used for each slide (these are shown in Supplementary Table 1). They show high homogeneity across various samples indicated by low standard deviations, and good linearity using a wide range of excitation powers (Fig. 2a,b right panel; Supplementary Fig. 2a). The emission spectra of the used fluorescent dyes were measured on cellular samples and on standard slides and were identical (Supplementary Fig. 2b). The stability of spectra and linearity of emission suggests that the slides are suitable for determining the S factors and the alpha. Reliability of the standard slides was further tested in an experiment, where 49 combinations of 14 standard slides were measured and a CV of 12.5% was achieved (Supplementary Table 2). As a further proof of the concept, the alpha factor was determined with BIIG2 and ab528 antibodies both on labeled A172 cells and using standard slides of the labeled antibodies (Fig. 2c). The alpha factor determined on standard slides showed smaller differences between the two antibodies, and a smaller error of measurement. One should also consider that antibodies with varying dye/protein ratios (DOL) may show altered binding affinity, so the DOL measured for the antibody solution and used in determining alpha may not match the average DOL of antibodies bound to cells^32^. This potential artifact can also be avoided using the antibody-based standard slides. Overall, these slides are an easy and cost-effective means for determining correction factors for FRET measurements, with the added benefit of being able to use the cheapest or most readily available antibody.

### Model systems for FRET measurements

**Figure 3:**
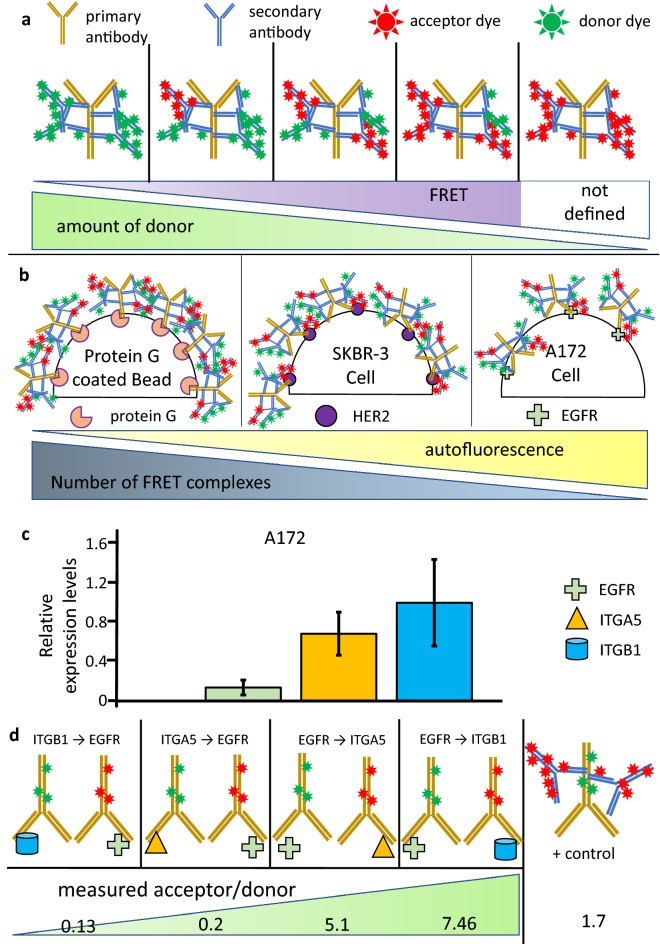
Model systems for testing pixel-by-pixel autofluorescence corrected FRET measurements. (**a**) Schematics of the molecular complexes covering a broad range of acceptor to donor dye ratio in the FRET model systems used. (**b**) Relative amount of the molecular FRET complexes (from panel **a**) on protein coated beads and on SKBR-3 and A172 cells, and their relation to autofluorescence intensity. (**c**) Relative expression levels of cell surface proteins on A172 cells. EGFR—EGF Receptor, ITGA5—Integrin α5, ITGB1: Integrin β1. Error bars represent ± SD, n = 6 images, minimum 30 cells. (**d**) Schematics of biologically relevant molecular complexes and the measured acceptor to donor dye ratios between them.

To test whether and under what conditions the PBP AF correction improves FRET measurements compared to average autofluorescence correction, a test system was constructed. In this system, a non-labeled primary antibody was used, and a secondary labeling mixing different ratios of donor and acceptor conjugated secondary antibodies. This way protein complexes with FRET efficiency ranging from low to very high values can be constructed (Fig. 3a). The primary antibodies were bound to either protein G coated beads, A172 cells or SKBR3 cells. Protein G beads have a high density of antibody binding sites in addition to a low autofluorescence, and can bind both trastuzumab (human) and ab528 (mouse) antibodies. SKBR3 cells express a high amount of HER2 (~ 800.000 per cell, bound by trastuzumab) and exhibit moderate autofluorescence. A172 cells have low expression of EGFR (~ 80.000 per cell, bound by ab528) and the highest autofluorescence of all targets used in this experimental setup. This way, all combinations of high and low FRET E with high and low autofluorescence and high and low intensities can be assessed (Fig. 3b). These samples can be measured both with a flow cytometer as well as in a microscope, allowing to compare the performance of the two approaches. For testing on biologically relevant FRET pairs, the A172 cell line expressing EGFR and integrins α5 (ITGA5) and β1 (ITGB1) was used. Here, the functional interaction of EGFR with either of the two integrin subunits^33,34^ yields varying acceptor/donor ratios and varying FRET E values (Fig. 3c,d). For FRET positive control, the donor conjugated primary antibody was labeled with the appropriate secondary antibody conjugated with the acceptor dye (Fig. 3d). The acceptor/donor ratios were very similar on all the three FRET positive control samples (Supplementary Fig. 3).

### FRET measurements on beads and cells with varying donor–acceptor ratios and varying autofluorescence

**Figure 4:**
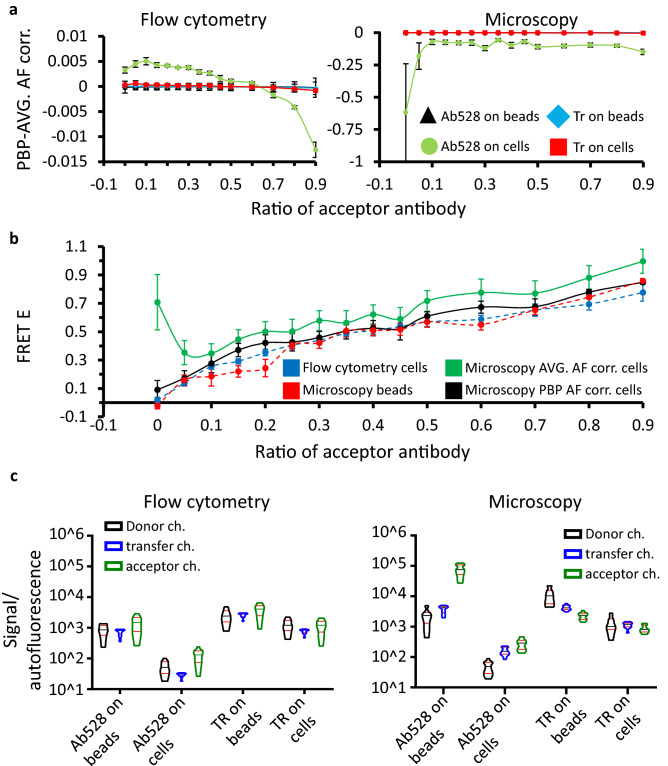
FRET measured on beads and cell suspensions with microscopy and flow cytometry. (**a**) Differences of the average and pixel-by-pixel autofluorescence correction, measured with flow cytometry and confocal microscopy on the control sample set. After gating, at least 1700 cells/5000 pixels remained for every plotted point. Error bars are ± SD. (**b**) FRET efficiencies measured with donor on the ab528 antibody, plotted against the ratio of acceptor-tagged antibody. (**c**) Fluorescence signals relative to autofluorescence, measured with flow cytometry and microscopy in all the FRET related channels. Data are shown on violin plots.

These FRET samples on beads and cells were measured with both flow cytometry and microscopy. The data obtained were evaluated with both AVG and PBP autofluorescence correction. AVG corrected FRET efficiency was subtracted from the PBP corrected value for each cell or bead in each sample, and the sample mean and SD of the differences were plotted against the ratio of acceptor-dye-conjugated to donor-dye-conjugated secondary antibodies bound to the primary targets. The difference between the AVG and the PBP correction methods was negligible, except for the high autofluorescence/low intensity sample (ab528 on A172 cells, Fig. 4a). This was especially prominent in the case of the microscope-based measurement, where the difference of FRET efficiencies was around 0.1, with a strongly increasing tendency at the lower acceptor to donor ratios (Fig. 4a, right panel). When comparing the actual FRET E versus acceptor-to-donor ratio plots (Fig. 4b), cell-based samples with low overall signal yield matching FRET efficiencies in flow cytometry and PBP corrected FRET microscopy, and these coincide with both PBP and AVG corrected FRET microscopy data from high intensity bead based samples. Only the low intensity cellular microscopy data evaluated using AVG correction diverge. This deviation from the presumed actual values is likely attributable to the lower signal per pixel. While using AVG correction necessarily over or under-corrects each pixel, this deleterious effect is most prominent at lower signal to noise ratios and the error is apparently propagated into the distribution of FRET efficiency. Using PBP AF correction does not improve the actual SNR, but at least makes away with the additional error introduced by correcting spatially uneven autofluorescence with a global constant, as demonstrated in Fig. 1c,d. Overall, the samples most sensitive to distortion by AVG correction are those with low labeling signal to autofluorescence ratio, as demonstrated for each measured fluorescence channel, both by flow cytometry and microscopy, in Fig. 4c. In our case, the lowest ratios were measured on EGFR-anchored ab528, binding the mixture of donor and acceptor tagged secondary antibodies, on A172 cells. One would consequently expect that in the case of using direct immunofluorescence to measure FRET between EGFR and other molecular species on the surface of these cells would cause even larger deviation from the actual values when using AVG correction.

### FRET between biologically relevant interacting molecules measured upon direct labeling, and indirect labeling-based positive controls

**Figure 5:**
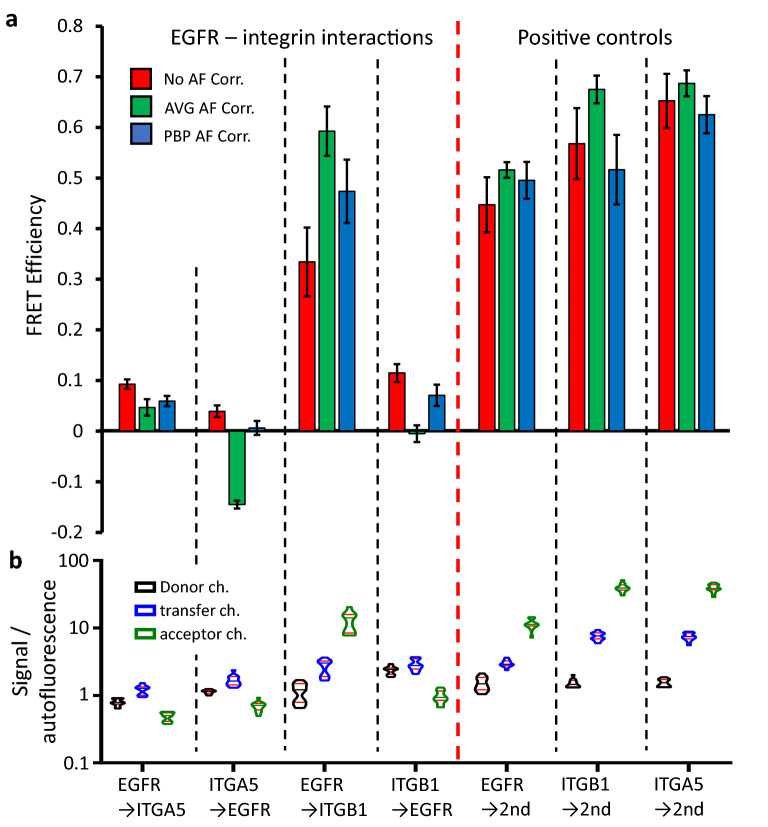
FRET measured with microscopy on adherent cells between biologically relevant molecules and on positive controls. FRET was measured between EGFR and ITGA5 or ITGB1 in both directions, as well as between primary and secondary antibodies bound to each of these molecular targets for positive controls. (**a**) FRET efficiencies calculated with no AF correction, average AF correction and pixel-by-pixel AF correction. Error bars are ± 95% confidence interval from 12 images, each containing a minimum of 5 cells. (**b**) Fluorescence signal relative to autofluorescence in the donor, transfer and acceptor channels, from the samples in (**a**). The means of all images in each category are shown as violin plots.

Therefore, we next measured the FRET efficiency between EGFR and ITGA5 or ITGB1 on A172 cells. Since these two integrins are expressed in a fivefold and eightfold ratio to EGFR, respectively (Fig. 3c), we measured FRET once with the donor and then with the acceptor label on EGFR to assess the influence of the acceptor/donor ratio. For these samples, the FRET efficiencies (Fig. 5a left panel) followed the trends of the measured acceptor/donor ratios (Fig. 3d), giving lower FRET E values for the cases where EGFR was labeled with the acceptor. The FRET efficiencies measured between EGFR and ITGA5 were generally low, with only minor differences between the non-corrected and PBP corrected values. AVG correction yielded negative average FRET E when ITGA5 was the donor, a strong indication that the AVG autofluorescence correction method can produce strong artefacts particularly when FRET E is low. The FRET efficiencies measured between the EGFR and ITGB1 were generally higher, indicating that steric positioning of the antibody labeling ITGB1 is more appropriate for the FRET process than that of ITGA5, even though these two integrins are expected to form heterodimers and thus could potentially interact with EGFR equally well. The AVG correction yielded a very high FRET efficiency when ITGB1 was the acceptor and 0 when it was the donor. At the same time, PBP correction yielded non-zero FRET efficiencies for both directions of energy transfer, with values at higher acceptor to donor ratios being higher, as would be expected from this stoichiometry. This underscores the inferiority of AVG autofluorescence correction to PBP correction. In fact, for all four samples, FRET E values gained with PBP autofluorescence correction were in the middle range, while those with no correction or with AVG correction were at the extremes, depending on the signal/autofluorescence ratio (Fig. 5b). Where the signal/autofluorescence ratio was higher in the donor channel, no correction gave the highest FRET values and AVG correction gave the lowest, and conversely, for the samples where the signal/autofluorescence values were higher in the acceptor channel, AVG correction resulted in higher values and no correction in lower FRET efficiencies.

We also measured and calculated the FRET efficiencies on positive control samples (Fig. 5a right panel). The measured acceptor/donor ratio was identical for all three samples (Supplementary Fig. 3), indicating that the differences in FRET efficiencies cannot be explained by the stoichiometry of the dyes. The AVG corrected FRET efficiencies followed the trends in signal/autofluorescence values in the FRET and acceptor channels while the non-corrected values did not follow a clear trend. PBP correction yielded FRET E values independent of the signal/autofluorescence ratios. Notably, it resulted in very similar FRET efficiencies for both mouse monoclonal primary antibodies (anti-EGFR and anti-ITGB1) and a different one for anti-ITGA5 which is a rat monoclonal antibody. Assuming that the complexes of donor labeled primary and acceptor labeled secondary antibodies are more similar when the same mixture of secondary antibodies is used, the difference between anti-rat and anti-mouse antibody-containing FRET complexes is acceptable. At the same time, the nearly identical values for the two anti-mouse secondary antibodies clearly indicate that PBP autofluorescence correction is superior to AVG correction and no correction.

**Figure 6:**
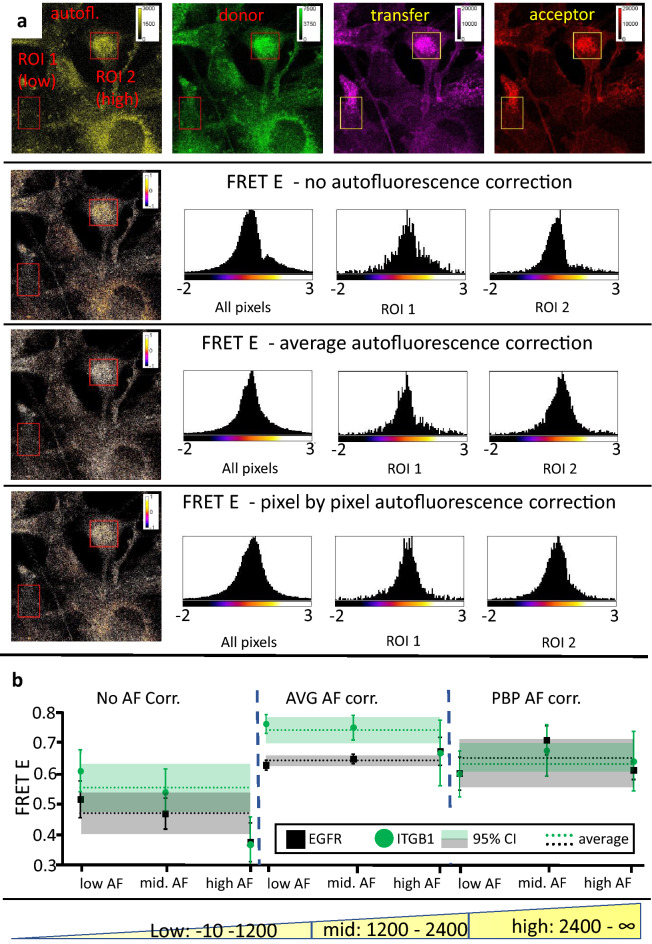
Effect of autofluorescence intensity on FRET calculated with different correction methods. (**a**) Sample images of the 4 fluorescent channel used for the FRET measurements (EGFR → 2nd antibody). ROI 1: low autofluorescence; ROI 2: high autofluorescence. Pixelwise distribution of FRET E for each correction method is shown for the whole image and for the low and high AF ROIs. (**b**) Pixels of 12 images each from samples prepared with EGFR and ITGB1 (low and high signal, respectively) were pooled and split into low (− 10–1.200), mid (1.200–2.400) and high (2.400–max) autofluorescence ranges. Average FRET E calculated with the three AF correction options (error bars representing ± 95% confidence interval) are plotted for each range and each sample. The global average for each sample and each correction method is also plotted (dashed lines) with its 95% confidence interval shown as bands of the same color.

For further analysis, the EGFR → second antibody sample was used. In Fig. 6a, two regions of interest (ROI), together with their FRET E histograms are shown; one with high and one with low autofluorescence. Histograms for the whole images are also displayed. PBP corrected histograms show a similar distribution for both the low and high AF regions and the whole image. However, no correction results in distortions for the high autofluorescence ROI, while the AVG correction generates similar anomalies at low autofluorescence intensities. To achieve a more quantitative view, all pixels of both the EGFR-based and ITGB1-based positive control samples were pooled and sorted into high, medium, and low autofluorescence ranges. Their FRET E histograms can be viewed in Supplementary Fig. 4. The mean values of the pooled pixels and their 95% confidence intervals obtained with no correction, with AVG and with PBP correction are shown in Fig. 6b. FRET efficiencies calculated with no AF correction are inversely proportional to autofluorescence. While in the low AF region they overlap with the PBP corrected range, with increasing autofluorescence they become increasingly under-corrected. In the case of AVG correction, the global mean values of FRET efficiencies are far apart for the low and high intensity samples, and in general are over-corrected. However, with PBP correction, the global averages of the two low and high intensity samples are very close and there is no tendentious influence of autofluorescence on the calculated FRET efficiency.

## Conclusions

In spite of important progress in accurately measuring FRET efficiency in autofluorescent samples^28^, a relatively straightforward method and software for AF correction that can be used with generally available equipment has not so far been available to the microscopy/biologist community. Here we provide an open source solution for pixel-by-pixel autofluorescence correction in microscopy FRET measurements and also describe the preparation and validation of a novel calibrations standard slide that allows for the unanimous determination of spectral spillover and instrument calibration factors independently of cellular autofluorescence. Based on these assets, we demonstrate, using various model systems, the benefits of pixel-by-pixel autofluorescence correction.

First, we have used a series of secondary antibody mixtures of varying proportions of acceptor and donor labeled molecules, encompassing a range of FRET efficiencies from 0 to maximum, and bound these to primary antibodies on cells and beads. We found, using flow cytometry measurements as a reference, that as the signal to autofluorescence ratio decreased below ~ 100, the microscopic determination of FRET E became increasingly inaccurate without pixel-by-pixel AF correction regardless of the actual FRET efficiency. Since in biological systems of interest signal to autofluorescence is often in this range, we next investigated such models, featuring pathologically important EGFR—integrin interactions^33,34^. Here we have shown that pixel-by-pixel AF correction increases the accuracy of FRET E calculation and abolishes the bias, which is inversely proportional to the signal to AF ratio.

Overall, we conclude that the pixel-by-pixel autofluorescence correction approach is particularly recommended for cases of highly variable cellular autofluorescence and for samples with low signal to autofluorescence ratio, which is typical for physiological expression levels. With the advent of fluorescence pathology scanners, the challenge of correcting for spatially varied autofluorescence becomes increasingly pressing, given that FRET measured in tissue sections by such devices could easily become, in the long term, a diagnostic approach^33,35^.

## Materials and methods

All reagents not specified otherwise were obtained from Sigma-Aldrich, St. Louis, MO, USA.

### Antibodies used

*Trastuzumab* (Herceptin®, Roche, binds HER2); *pertuzumab* (Perjeta®, Roche, binds HER2); *TS2/16.2.1* mouse monoclonal produced in-house from hybridoma (ATCC Cat# HB-243, binds integrin β1); *BIIG2* rat monoclonal produced in-house from hybridoma (DSHB Antibody Registry ID: AB_528155, binds α5 integrin); *ab528* mouse monoclonal produced in-house from hybridoma (ATCC Cat# HB-8509 binds: epidermal growth factor receptor EGFR);*Alexa Fluor 546 conjugated goat anti-Mouse IgG* (H + L), highly cross-adsorbed (ThermoFisher cat# A-11030); *Alexa Fluor 647 conjugated Goat anti-Mouse IgG* (H + L) highly cross-adsorbed (ThermoFisher cat# A-21235); *Alexa Fluor 546 conjugated Goat anti-Human IgG* (H + L) highly cross-adsorbed (ThermoFisher cat# A-21089); *Alexa Fluor 647 conjugated Goat anti-Human IgG* (H + L) highly cross-adsorbed (ThermoFisher cat# A-21445);*Alexa Fluor 647 conjugated Goat anti-Rat IgG* (H + L) highly cross-adsorbed (ThermoFisher cat# A-21247).

### Preparation of standard slides

Monoclonal antibodies (trastuzumab; pertuzumab; ab528; TS2) were labeled with Alexa Fluor 546 or Alexa Fluor 647 Succinimidyl ester (ThermoFisher Cat# A20002 & A20006) according to manufacturer’s recommendations, then purified on a Sephadex G50 column (Cat# 9048-71-9). The resulting protein and dye content were measured with a NanoDrop ND-1000 spectrophotometer. 5 µl conjugated antibody solution was diluted in 45 µl solvent comprising of 0.3 m/m% BSA (VWR Chemicals cat# 0332-100G) and 70% Mowiol antifade comprising 0.1 M TrisHCl, pH 8.5, 25% (w/v) glycerol, and 10% Mowiol 4-88, Polysciences, Warrington, PA, USA in PBS. 30 µl of this diluted antibody was pipetted on a slide and covered with acetone-cleaned 12 mm diameter round glass coverslips (0.17 mm thickness). Samples were dried overnight at room temperature and stored at 4 °C afterwards. For determining the instrument background, a non-fluorescent slide was made by replacing the 5 µl antibody conjugate with the same volume of 1 m/m% BSA in PBS.

### Cell culture

A172 glioblastoma (ATCC Cat# CRL-1620) and SKBR3 breast cancer (ATCC Cat# HTB-30) cells were cultured in DMEM supplemented with 10% FBS, at 37 °C with 5% CO_2_ in a humidified incubator for 2 days in T75 cell culture flasks or on sterilized 12 mm diameter round glass coverslips (0.17 mm thickness).

### Fluorescent labeling in suspension

Cells grown in a T75 flask were rinsed with 5 ml HEPES buffer supplemented with 4 mM glucose, then treated with 0.5 g/dm^3^ trypsin (Cat# 27250018) and 0.2 g/dm^3^ EDTA (Cat# 15576028) in 1 ml sterile-filtered PBS for 5 min at 37 °C. The detached cells were collected and washed for 5 min at 4 °C in HEPES buffer supplemented with 4 mM glucose at 200 g in a standard 15 ml tube. The supernatant was removed, and the pellet was resuspended and fixed in 1 ml 1% formaldehyde in HEPES buffer for 15 min at room temperature. The cells were washed again for 5 min in HEPES buffer at room temperature. The A172 and SKBR3 cells were labeled for 30 min at room temperature with 20 µg/ml final concentration of ab528 and trastuzumab, respectively, then washed with HEPES buffer supplemented with 4 mM glucose and centrifuged at 200*g*. For secondary fluorescent labeling, A172 cells were labeled with different ratios of goat anti mouse antibodies conjugated with Alexa Fluor 546 or Alexa Fluor 647 at a total concentration of 10 µg/ml. SKBR3 cells were labeled in the same manner, except goat anti human antibodies were used. After 30 min of incubation at room temperature, the cells were washed as previously described and the pellets were resuspended in 150 µl HEPES buffer containing 1% formaldehyde. Non-labeled controls were made using the same protocol but omitting the antibodies.

The labeling of the Protein G coated beads (Bangslabs Protein G Antibody Binding Beads cat# 554) followed a similar protocol but without applying the first fixation step. The primary antibody concentrations were 100 µg/ml to achieve saturation concentration (Supplementary Fig. 5).

20 µl of every sample (cells and beads) was mixed with 20 µl of Mowiol antifade. 30 µl of this mixture was pipetted on a Superfrost glass slide and covered with acetone-cleaned 12 mm diameter round glass coverslips (0.17 mm thickness). Slides were dried overnight at room temperature and stored at 4 °C.

The slides were imaged with a Zeiss LSM 880 laser scanning microscope. The remaining unmounted sample suspensions were measured with an ACEA NovoCyte RYB flow cytometer.

### Fluorescent labeling of adherent cells

A172 cells cultured on 12 mm diameter round glass coverslips were removed from the medium and placed on an ice-cold parafilm-coated metal surface and washed with HEPES buffer supplemented with 4 mM glucose. The cells were fixed for 15 min at 4 °C with 1% formaldehyde in HEPES buffer, then washed 3 times with HEPES buffer (for 2, 3, and 7 min) at room temperature. All consecutive steps were done at room temperature. For direct labeling, cells were labeled with donor and acceptor labeled antibodies, each at 20 µg/ml final concentration in HEPES buffer containing 2% BSA for 30 min, using the following combinations: ab528-Alexa Fluor 546 + BIIG2-Alexa Fluor 647; ab528-Alexa Fluor 647 + BIIG2-Alexa Fluor 546; ab528-Alexa Fluor 546 + TS2-Alexa Fluor 647; ab528-Alexa Fluor 647 + TS2-Alexa Fluor 546. For indirect labeling, primary antibodies ab528-Alexa Fluor 546, BIIG2-Alexa Fluor 546 and TS2-Alexa Fluor 546 were used in the same manner, at 20 µg/ml final concentration. After this labeling step, cells were washed again 3 times (for 2, 3 and 7 min) with HEPES buffer. In the case of indirect labeling this was followed by a similar labeling cycle using 10 µg/ml Alexa Fluor 647 conjugated secondary antibodies suitable for the primer antibody diluted in HEPES containing 2% BSA for 30 min. The final wash was followed by fixation in 1% formaldehyde in HEPES buffer for 15 min and mounting on Superfrost glass slides with Mowiol. Single labeled and unlabeled controls were also prepared for determining correction factors and emission spectra.

### Laser scanning confocal microscopy

All imaging was done with a confocal laser scanning microscope (LSM 880, Carl Zeiss GmbH, Jena, Germany), using a 40× C-Apochromat water immersion objective (NA = 1.2, item no.: 421767-9971). The pinholes were set to 1 µm slice thickness, and the pixel size to 100 nm (Nyquist-based optimal sampling). For spectrally resolving the autofluorescence, the emission of non-stained cells was resolved onto a 32 element GaAsP array of the microscope with a step size of 8.92 nm, using sequentially 488, 543, and 633 nm excitation. The same data acquisition strategy was employed for verifying the spectral stability of donor and acceptor emission across standard slides and labeled cells.

For the FRET measurements, the microscope was operating in line scanning mode and all the fluorescent channels were detected by the 32 channel GaAsP detector of the instrument. In the autofluorescence channel, the excitation was 488 nm and the detection range was 499–535 nm. The donor and the transfer channels were excited simultaneously with 543 nm excitation and detected between 553–615 nm and 651–695 nm, respectively. The acceptor channel was excited at 633 nm and detected in the 651–695 nm range.

### Flow cytometry

Flow cytometric fluorescence and FRET measurements were conducted with a NovoCyte RYB cytometer. For autofluorescence, the instrument’s predefined FITC-H channel was used (488 nm excitation and 515–545 nm detection); the donor channel was PE-H (561 nm excitation and 576–596 nm detection); the transfer channel was PE-Cy5 mPlum-H (561 nm excitation and 650–670 nm detection); and the acceptor channel APC-H (640 nm excitation and 650–670 nm detection). For evaluation, the gating and correction factor determination was made in FCS Express v7. The gated FRET samples were exported to CSV, the FRET calculation formulas (see equations) were applied in MS excel, then the newly created FRET list mode data imported to FCS Express for visualization.

### Image analysis

All image analysis was done using Fiji (https://fiji.sc/)^36^. A plugin previously created in our lab for calculating conventional spectral spillover-corrected quantitative (3-filter) FRET (RiFRET^16^), has been re-written to include pixel-by-pixel autofluorescence correction, as well as to offer fully automatic processing of larger image sets and to be compatible with ImageJ 2.0. The source code is available on GitHub (https://github.com/CellMoTher/RiFRET) and the plugin is available via the "FRET Imaging" update site in Fiji.

## Supplementary Information

Supplementary Information.

## Data Availability

The datasets generated and analysed in this study are available from the corresponding author upon reasonable request. No data pertinent to public databases have been generated.
