# Supplementary material for: Pixel-by-pixel autofluorescence corrected FRET in fluorescence microscopy improves accuracy for samples with spatially varied autofluorescence to signal ratio
Source: Sci Rep. 2023 Feb 20;13:2934. doi: 10.1038/s41598-023-30098-w (PMC9941493; doi:10.1038/s41598-023-30098-w)
Supplement: Supplementary file 1 — Supplementary Information. [file 41598_2023_30098_MOESM1_ESM.pdf]

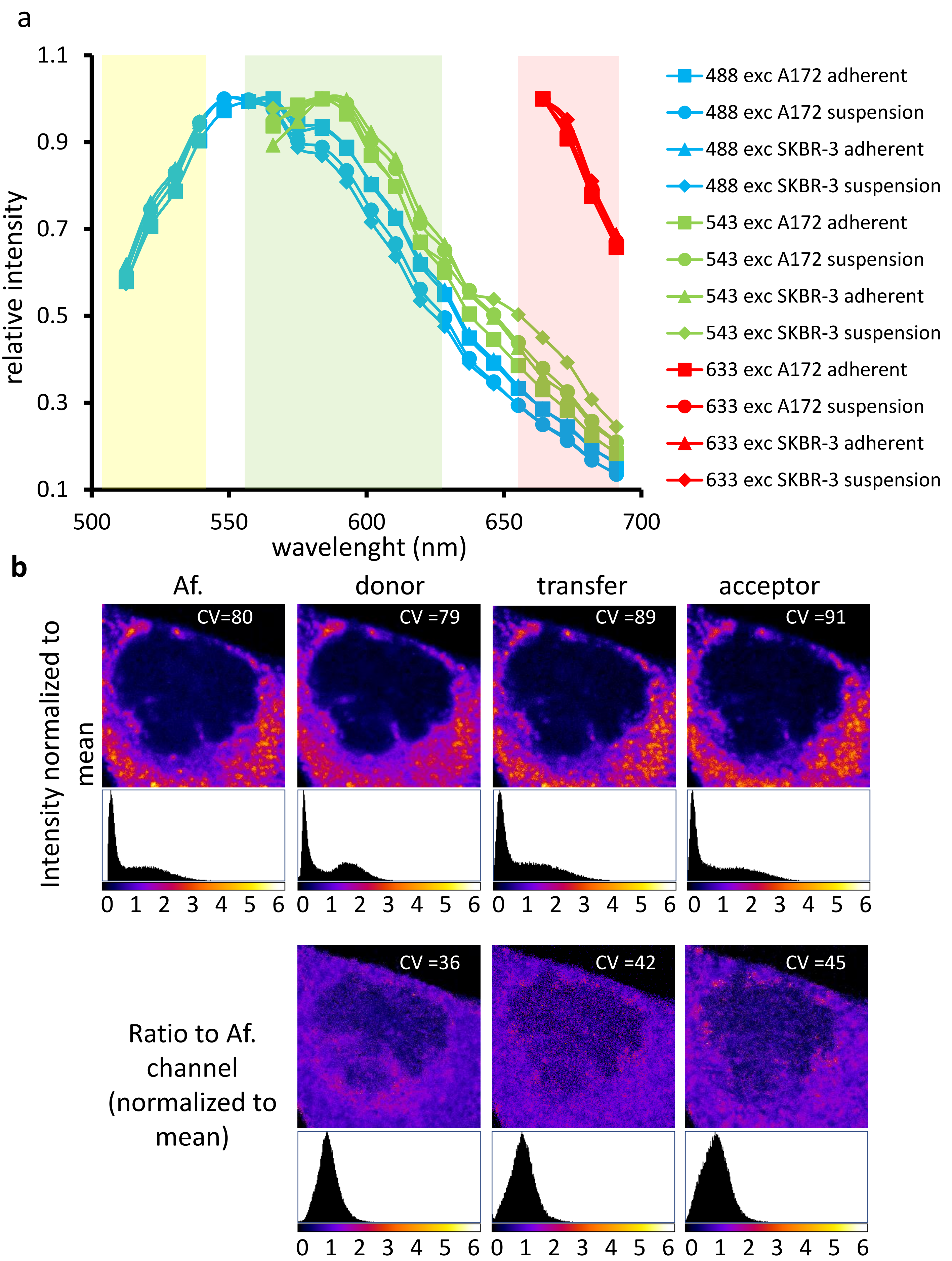
a
relative intensity
1.1
0.9
0.7
0.5
0.3
0.1
500
550
600
650
700
wavelenght (nm)
488 exc A172 adherent
488 exc A172 suspension
488 exc SKBR-3 adherent
488 exc SKBR-3 suspension
543 exc A172 adherent
543 exc A172 suspension
543 exc SKBR-3 adherent
543 exc SKBR-3 suspension
633 exc A172 adherent
633 exc A172 suspension
633 exc SKBR-3 adherent
633 exc SKBR-3 suspension
b
Af.
donor
transfer
acceptor
Intensity normalized to mean
CV=80
CV =79
CV =89
CV =91
0 1 2 3 4 5 6
Ratio to Af. channel (normalized to mean)
CV =36
CV =42
CV =45
0 1 2 3 4 5 6

Supplementary fig. 1.
Characterization of the autofluorescence of adherent and suspended cells.

a: Autofluorescence emission spectra of A172 and SKBR-3 cells measured with a spectral detector upon 488; 543 and 633 nm excitation. The typical emission channels of a FRET measurement are represented as colored bands. Traces for cells grown on coverslips and for cells mounted in suspension after trypsinization are both plotted.

b: Mean normalized images and intensity histograms of a nonlabelled A172 cell in the autofluorescence (Af.), donor, transfer, and acceptor channels, and their ratios to the autofluorescence channel.

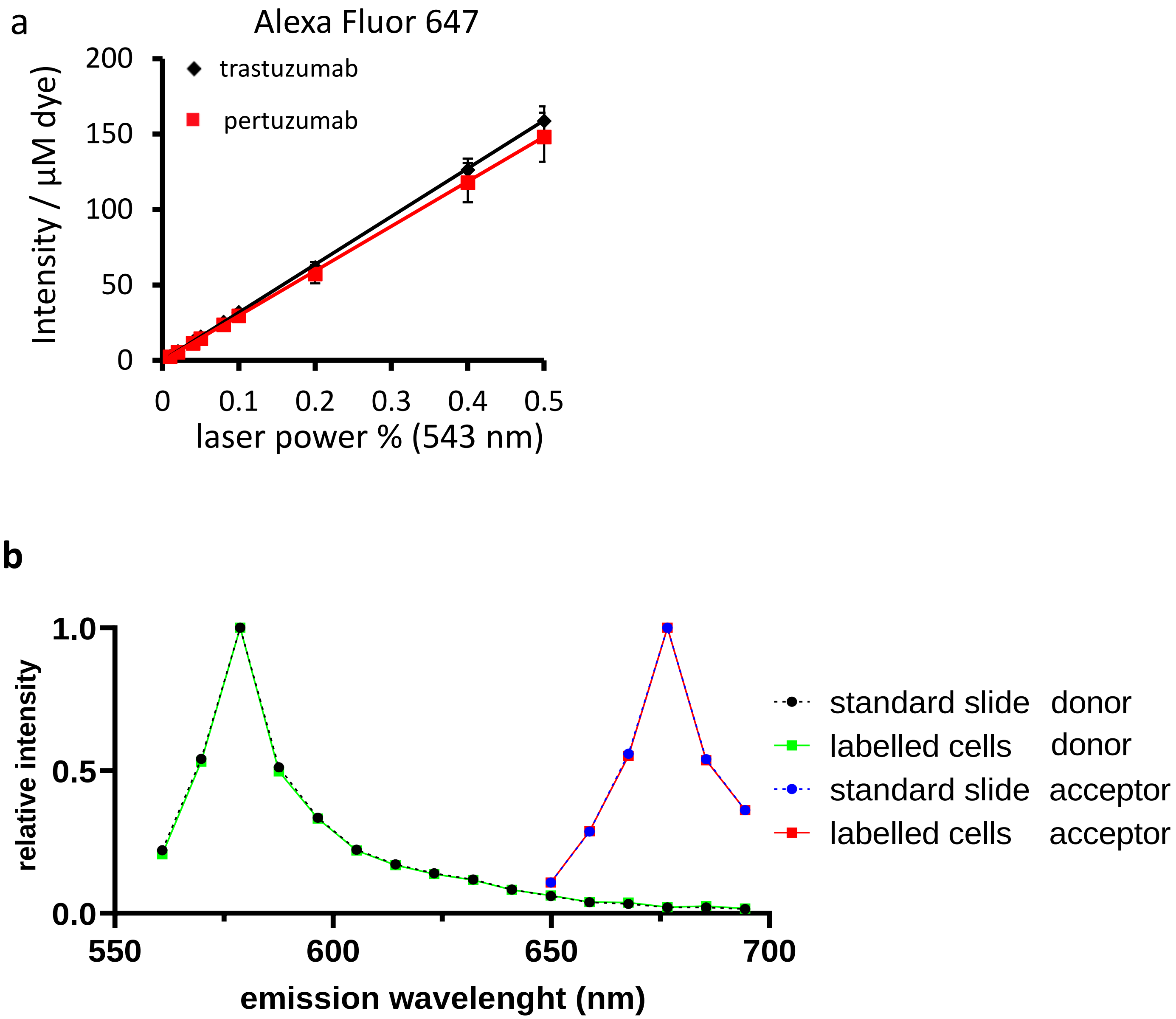
a
Alexa Fluor 647
trastuzumab
pertuzumab
Intensity / µM dye)
laser power % (543 nm)
0
50
100
150
200
0
0.1
0.2
0.3
0.4
0.5
b
relative intensity
emission wavelenght (nm)
0.0
0.5
1.0
550
600
650
700
standard slide donor
labelled cells donor
standard slide acceptor
labelled cells acceptor

Supplementary fig. 2.
Linearity at low laser powers and spectral reliability of acceptor standard slides

a: Average intensities of Alexa Fluor 647 conjugated trastuzumab and pertuzumab, plotted against 543 nm excitation laser powers, normalized to dye concentration. Error bars represent ±SD of n=5 images.

b: Emission spectra of Alexa Fluor 546 (donor) and Alexa Fluor 647 (acceptor) on labbeled SKBR-3 cells and on standard slides, measured with a spectral detector using 543 and 633 nm excitation, respectively.

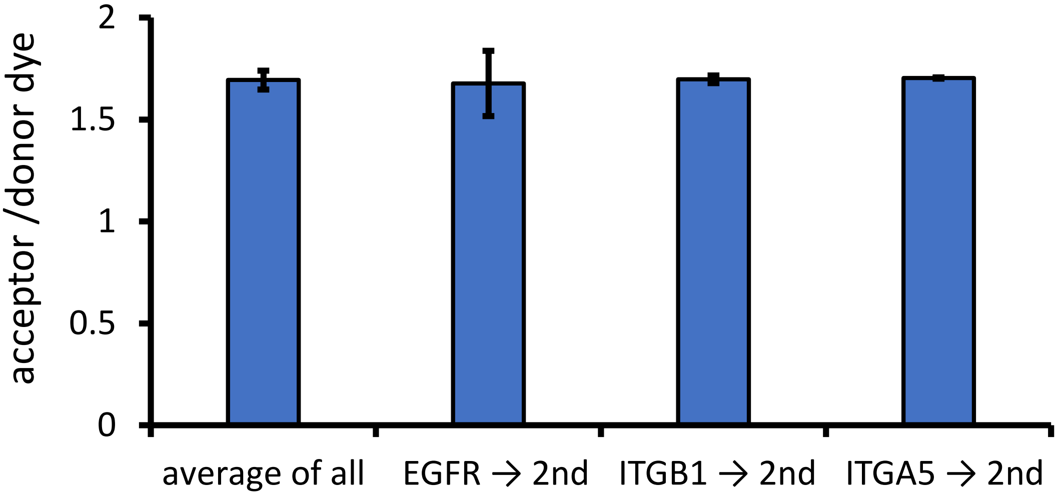
2
1.5
1
0.5
0
acceptor /donor dye
average of all
EGFR → 2nd
ITGB1 → 2nd
ITGA5 → 2nd

Supplementary fig. 3.
Ratios of acceptor to donor dyes measured on cell-based FRET positive controls.

EGFR, ITGB1 and ITGA5 on A172 cells were labeled with the appropriate donor-conjugated primary antibody followed by acceptor conjugated secondary antibody. Ratio of acceptor to donor fluorescence was calculated from 12 microscopic images (at least 5 cells per image) for each label after correction for donor quenching and sensitized acceptor emission. Error bars represent ± 95% confidence interval.

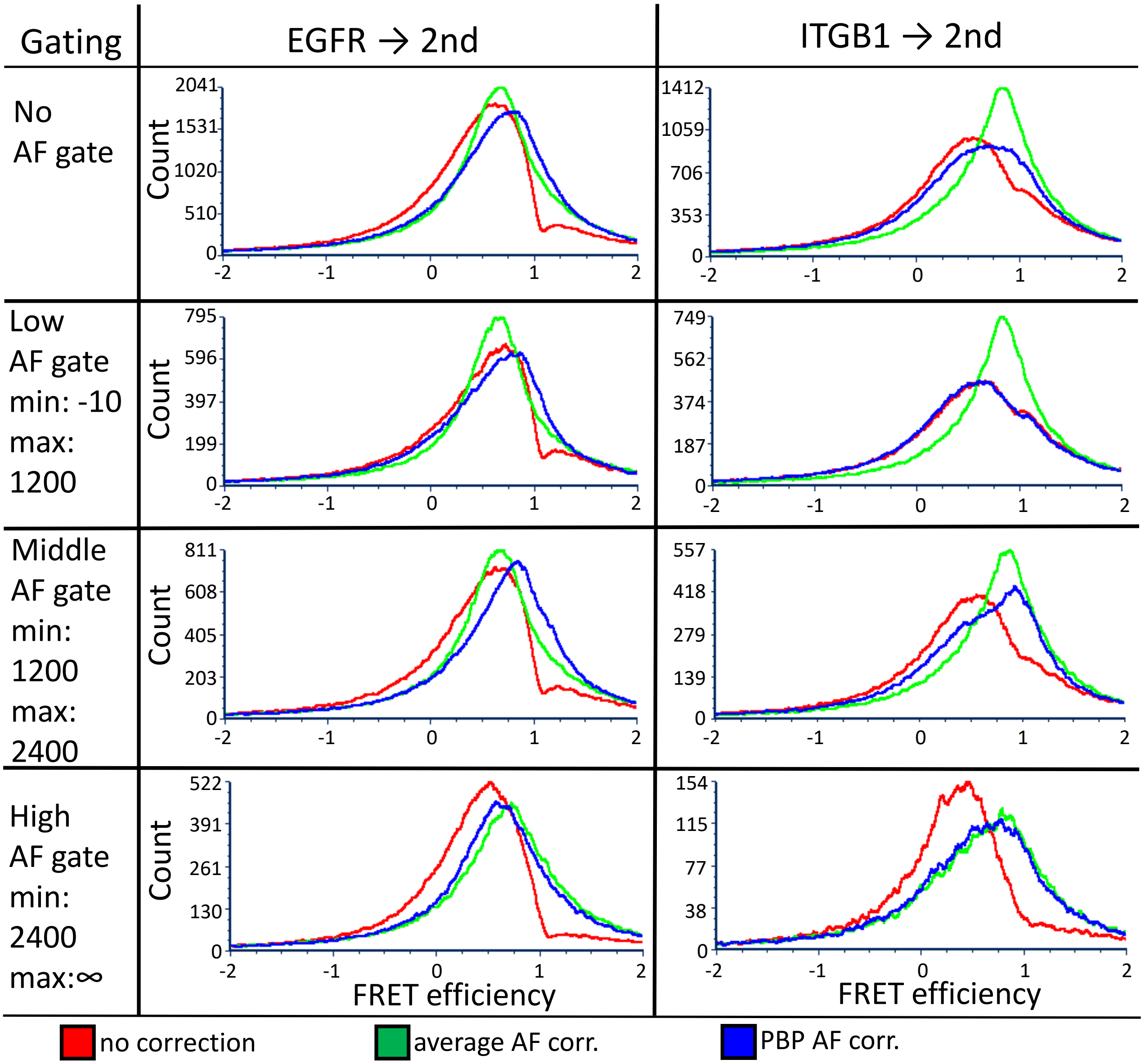
Gating
EGFR → 2nd
ITGB1 → 2nd
No AF gate
Low AF gate min: -10 max: 1200
Middle AF gate min: 1200 max: 2400
High AF gate min: 2400 max:∞
Count
2041
1531
1020
510
0
1412
1059
706
353
795
596
397
199
749
562
374
187
811
608
405
203
557
418
279
139
522
391
261
130
154
115
77
38
-2
-1
0
1
2
FRET efficiency
no correction
average AF corr.
PBP AF corr.

Supplementary fig. 4.
Effect of autofluorescence intensity on FRET histograms calculated with different correction methods.

Pixels of 12 images each from samples prepared with EGFR and ITGB1 (low and high signal, respectively) were pooled and the pixels (>1.2 million per sample) split into low (-10—1.200), mid (1.200—2.400) and high (2.400—max) autofluorescence ranges. FRET E histograms calculated with the three AF correction options are plotted for all the pooled pixels and for each AF range, for both samples.

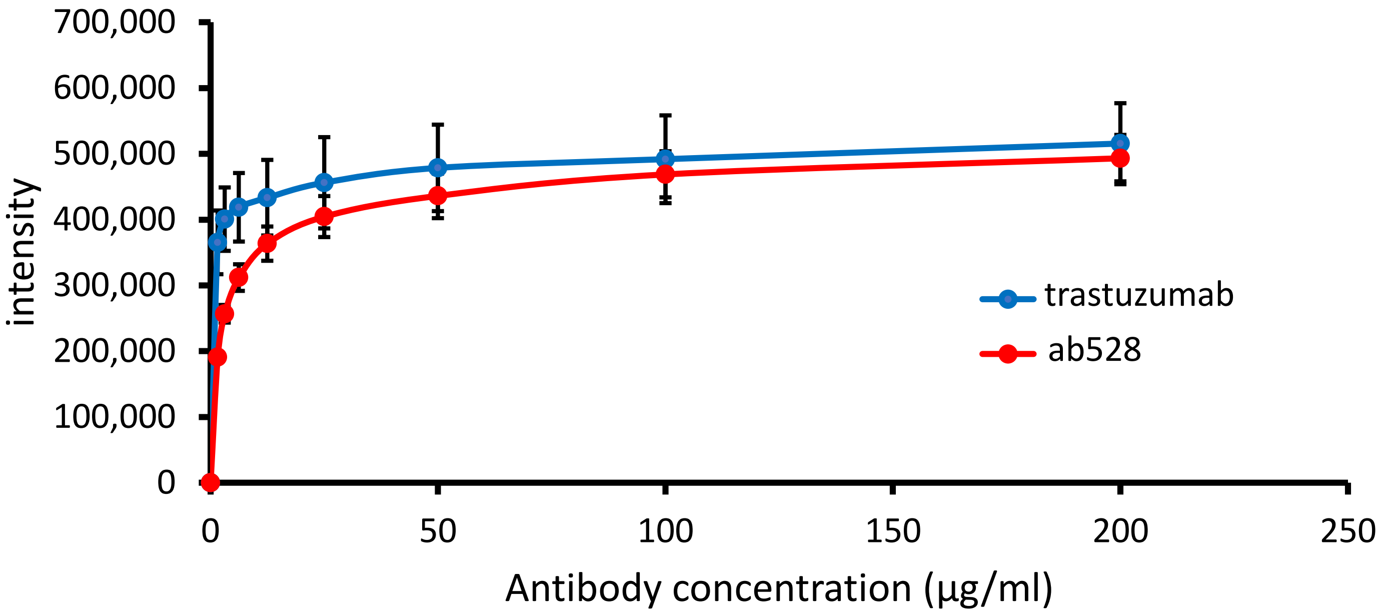

700,000
600,000
500,000
400,000
300,000
200,000
100,000
0
intensity
0
50
100
150
200
250
Antibody concentration (µg/ml)
trastuzumab
ab528

Supplementary fig. 5.

Dose-response curves of protein G coated beads labeled with fluorescently conjugated antibodies.

Mean fluorescence intensity (arbitrary units) of at least ~ 1000-2000 beads per data point is plotted against trastuzumab or ab528 antibody concentration. Error bars are ± SD.

Supplementary tables

Supplementary table 1.

antibody	Alexa Fluor 546					Alexa Fluor 647				
	dye concentration (µM)			protein mg/ml	DOL	dye concentration (µM)			protein mg/ml	DOL
	average	SD	CV			average	SD	CV		
ab528	13.077	0.308	0.024	0.979	1.912	12.222	0.174	0.014	0.728	2.472
Trastuzumab	12.737	0.281	0.022	1.033	1.595	12.533	0.019	0.002	0.989	1.782
Pertuzumab	14.356	0.318	0.022	0.868	2.588	13.880	0.032	0.002	0.996	3.825

Characteristics of antibodies used for preparing standard calibration slides. Dye concentration measured with nanodrop ND 1000 (3 technical replicates), protein concentration and degree of labeling (DOL, molar ratio of dye to protein) are shown.

Supplementary table 2.

alpha								
		Alexa Fluor 546						
		Tr. 1	Tr. 2	Tr. 3	Pr. 1	Pr. 2	Pr. 3	ab528
Alexa Fluor 647	Tr. 1	0.305	0.393	0.356	0.347	0.347	0.360	0.370
	Tr. 2	0.277	0.356	0.323	0.314	0.314	0.326	0.335
	Tr. 3	0.310	0.399	0.361	0.352	0.352	0.366	0.376
	Pr. 1	0.276	0.355	0.321	0.313	0.313	0.325	0.334
	Pr. 2	0.301	0.387	0.350	0.342	0.342	0.355	0.364
	Pr. 3	0.239	0.308	0.279	0.272	0.272	0.282	0.290
	ab528	0.236	0.303	0.275	0.268	0.268	0.278	0.286

Average	0.322
SD	0.040
CV	0.125
n	49

Alpha calibration factors and their descriptive stastistics calculated from combinations of seven donor and seven acceptor conjugated antibody-based standard slides. Slides were imaged under identical circumstances in a confocal lase scanning microscope.
